# Effects of early language, speech, and cognition on later reading: a mediation analysis

**DOI:** 10.3389/fpsyg.2013.00586

**Published:** 2013-09-03

**Authors:** Vanessa N. Durand, Irene M. Loe, Jason D. Yeatman, Heidi M. Feldman

**Affiliations:** ^1^Division of Neonatal and Developmental Medicine, Department of Pediatrics, Stanford School of MedicinePalo Alto, CA, USA; ^2^Vision Imaging Science and Technology Lab, Department of Psychology, Stanford UniversityStanford, CA, USA

**Keywords:** mediation, cognition, language, speech, reading, comprehension, preschool, vocabulary

## Abstract

This longitudinal secondary analysis examined which early language and speech abilities are associated with school-aged reading skills, and whether these associations are mediated by cognitive ability. We analyzed vocabulary, syntax, speech sound maturity, and cognition in a sample of healthy children at age 3 years (*N* = 241) in relation to single word reading (decoding), comprehension, and oral reading fluency in the same children at age 9–11 years. All predictor variables and the mediator variable were associated with the three reading outcomes. The predictor variables were all associated with cognitive abilities, the mediator. Cognitive abilities partially mediated the effects of language on reading. After mediation, decoding was associated with speech sound maturity; comprehension was associated with receptive vocabulary; and oral fluency was associated with speech sound maturity, receptive vocabulary, and syntax. In summary, all of the effects of language on reading could not be explained by cognition as a mediator. Specific components of language and speech skills in preschool made independent contributions to reading skills 6–8 years later. These early precursors to later reading skill represent potential targets for early intervention to improve reading.

## Introduction

The ability to read well confers many educational and occupational advantages. Children with poor reading skills in early elementary school rarely catch up to children with strong reading skills (O'Connor et al., 2005). For this reason, identifying early precursors or predictors of later reading skills is important because such precursors may be appropriate targets for early intervention aimed at improving long-term reading outcomes. Many studies that assess language or cognitive skills in the preschool period in relation to reading at school age consider only a single ability as the predictor variable. Most studies evaluate early skills in relation to broad reading ability without separating single word reading and comprehension. In addition, previous studies rarely conduct assessments of children before the age of 4 years or after the 2nd grade (National Center for Family Literacy, 2008; Thomson and Hogan, 2010). The overall goal of the current longitudinal secondary analysis was to evaluate multiple measures of language, speech, and cognitive skills at age 3 years in relation to multiple reading outcomes at age 9–11 years.

Reading is comprised of two essential component skills—single word reading or decoding and comprehension (Hoover and Gough, 1990; Jenkins et al., 2003). Decoding refers to the ability to translate written letters into speech sounds and to recognize written single words accurately. It is assessed by having a child read single words or pseudowords. Comprehension refers to the ability to extract meaning from written words and sentences. It is assessed by having the child answer questions about sentences and passages after reading them. Another domain of reading is oral reading fluency, defined as the speed at which an individual can produce spoken words from written words. It is assessed by calculating the speed and accuracy of the oral output (Fuchs et al., 2001). Though oral reading fluency correlates with reading comprehension (Kim et al., 2012), when students begin to shift from learning to read to reading to learn in later elementary school, deficits in oral reading fluency often emerge, even in the absence of reading comprehension deficits (Chall, 1983).

Many studies, using different methodologies, document that early language skills are associated with later reading skills (Rayner et al., 2001; Shaywitz et al., 2008). Studies of children with speech and language delays or deficits in the preschool period find that these children are at high risk for reading disability at later ages (Scarborough and Dobrich, 1990; Catts et al., 2002; Rescorla, 2002). For example, Bishop and Adams (Bishop and Adams, 1990) documented that children with language impairments at age 5.5 years had reading difficulties as well as oral language impairments at age 8.5 years of age. Scarborough (1990) found that three of four preschool children with language delays showed reading disorders at age 8 years, even after they caught up to peers in language skills. However, language and reading disorders are not completely overlapping; Catts et al. (2005) classified specific language impairment and reading disorder as separate and co-existing conditions with a 17% overlap.

Longitudinal studies of children across the spectrum of language abilities have also found associations of preschool language skills with later reading. For example, in a study of 74 low-income children attending Head Start, Dickinson and Tabors (2001) found a positive association between language measures obtained at 3 years of age and measures of both decoding and reading comprehension obtained at 14 years of age. In a larger study of 604 children, Catts et al. (1999) used regression modeling to show that language and phonological processing in kindergarten accounted for unique variance in later reading achievement. In their sample, language abilities measured in kindergarten predicted 49% of the variance in reading comprehension and 33% of the variance in word recognition in second grade. In a follow-up study, students with poor language comprehension in kindergarten remained deficient in spoken language skills measured in eighth grade, and the language difficulties continued to contribute to poor reading comprehension (Catts et al., 2006). Storch and Whitehurst (2002) used structural equation modeling to demonstrate that spoken language skills did not have a direct effect on reading, but rather had a significant effect on phonological skills that in turn affected decoding. However, they found that as children grew older, spoken language skills had a direct effect on reading comprehension skills. Following up on these observations, a longitudinal study of 1137 children conducted by the National Institute of Child Health and Human Development (NICHD) assessed the relationship between oral language skills at 36 months and reading abilities in 3rd grade (NICHD Early Child Care Research Network, 2005). The findings of this study supported the position that the child's early language skills contributed to decoding and reading comprehension both directly and indirectly by influencing phonological awareness, which in turn affected later reading abilities.

Language is not a unitary construct but rather a complex ability comprised of multiple component skills. These components are usually correlated in high-functioning adults and children but may be dissociated in individuals with various clinical conditions, such as specific language impairment (Johnson et al., 1999). The first broad subdivision is between receptive language, the ability to understand, and expressive language, the ability to produce words, sentences, discourse, and conversation. In early childhood, receptive language is typically measured by having the child point to the picture within a field of pictures that matches a given word or phrase. Expressive language may be assessed from recorded samples of spontaneous conversation during a play activity. Vocabulary is defined as the words of the language. Strong vocabulary skills may be linked to later reading ability because they help children to decode words that they have never seen in text or help them to comprehend what they are reading because they understand the surrounding context (Muter et al., 2004; Cain and Oakhill, 2011). In addition, syntax or grammar may be associated with later reading because children with strong syntactic skills may be able to comprehend context even when they have difficulty with specific decoding. In addition, syntactic difficulties are associated with specific language impairments (Bishop and Adams, 1990) that are associated with poor reading.

Speech is the verbal output of the language system. Speech sound maturity may influence later reading outcomes since the ability to read requires linking graphemes to speech sounds. In addition, rates of speech development correlate with rates of language development that in turn are associated with reading (Tsao et al., 2004). As positive evidence for the association, one study found that kindergarten-aged children who were reading-delayed made more typical and atypical speech errors than children who read at grade level (Foy and Mann, 2012). Kindergarteners who scored poorly on tests of speech sound production exhibited greater deficits in reading ability measured in the 1st and 2nd grade, even though children's vocabulary skills were in the average range (Overby et al., 2012).

Many studies also confirm an association between cognitive or intellectual abilities and reading skills. In a meta-analysis of 34 studies, the median correlation between IQ and reading abilities was approximately 0.45 (Hammill and McNutt, 1981). Stanovich et al. (1984) found that correlations between intelligence scores and reading generally increased with age, from 0.33 in first grade to 0.56 in fifth grade. Ferrer and colleagues also documented that reading abilities in children grade 1–12 were associated with cognition, but in this study, the association was strongest during 1st through 3rd grade (Ferrer et al., 2007). In a later study, Ferrer and colleagues also found that in children who had developmental dyslexia, cognition and reading ability developed independently from each other (Ferrer et al., 2010).

Early language development is also highly associated with cognitive abilities (Walker et al., 1994). Researchers have shown that language structure is related to domains of cognition that require abstract and fluid thought (Astington and Jenkins, 1999; Boroditsky, 2001). Delays in the development of language are often the presenting concern for children who ultimately develop learning or intellectual disabilities. A longitudinal study of children assessed at 3, 5, and 7 years in New Zealand (Silva et al., 1983) showed that children with receptive, expressive, and general language delays had a significantly higher prevalence of intellectual disabilities or reading difficulties at age 7 years than control children.

Few previous studies have considered which early language and/or speech abilities are associated with which specific reading components, i.e., decoding, reading comprehension, and/or oral reading fluency. The literature is also unclear on whether these early language and speech abilities are independently associated with later reading components or whether the associations may be mediated by early cognitive abilities. To address these questions, a large sample must be followed longitudinally over a long span of development and comprehensively assessed at early and late ages. We had available to us such a longitudinal data set from a large study of possible effects of early-life otitis media (i.e., middle-ear infection and effusion) on speech, language, cognition, and academic skills (Paradise et al., 1997, 2001, 2003, 2005, 2007) Within this data set was a diverse representative sample of children whose experience with otitis media ranged from none at all to just below the study threshold for randomization. We focused on this group as representative of a diverse and healthy population. Advantages of this data set to answer questions regarding the associations between early language and speech abilities and later reading include its large sample size, socioeconomic diversity, minimal attrition of subjects over time, and comprehensive data collection during preschool and school age.

The first goal of the study was to identify which, if any, of a set of three measures of language skills and one measure of speech sound maturity assessed at age 3 years would be associated with any of the three components of reading abilities—single word reading (decoding), comprehension, and oral reading fluency—in the same children at ages 9–11 years. Based on the extant literature, we hypothesized that associations between early and later skills would be positive, though we did not know whether all measures of language and speech would be associated with all components of reading. The second goal was to determine whether language and speech skills at age 3 years would be associated with cognitive abilities at age 3 years and whether cognitive abilities at age 3 years would be associated with the three components of reading at age 9–11 years. We hypothesized that these associations would all be positive correlations. The third goal of the study was to determine if cognitive abilities in the preschool period mediated the effects of language and speech on later reading skills. We hypothesized that language and speech skills would not be fully mediated by cognition, indicating that aspects of language and/or speech would make independent contributions to reading skills, even if the language and speech abilities were associated with cognitive skills.

The rational for choosing a mediation analysis relates its utility in planning prevention and intervention studies (MacKinnon et al., 2007). We know that language influences cognitive skills, but we do not think that this association indicates a simple, unidirectional causal mechanism. Rather we believe that both language and cognition influence each other and are impacted by other critical variables, including neurological integrity and the quality of environmental input. The value of mediation in this study is that it indicates whether preschool language and speech skills make independent contributions to school-aged reading abilities. If all of the associations between language and speech in preschool on later reading would be mediated through cognitive ability, then cognition might be a sufficient target for early intervention to improve reading outcomes. A useful intervention to improve reading might be an enriched preschool environment that stimulates thinking and problem solving skills. However, if language and speech make independent contributions to reading skills, then these early skills may also need to be targeted in early interventions to improve later reading. We hypothesized that language or speech skills would remain associated with reading abilities even when early cognitive skills were evaluated as a mediator.

## Methods

### Participants

Participants for this secondary data analysis were part of a larger study of the developmental outcomes of otitis media and its treatment. The details of participant recruitment and methods are extensively detailed in previous studies (Paradise et al., 1997, 2001, 2007).

This report analyzed a group of 241 children called the “association” cohort who were followed from birth to age 9–11 years. This group represented the demographics of the study sample as a whole and included children who exhibited a spectrum of ear infection from no middle ear effusion to those with too short a duration of effusion to warrant consideration of tympanostomy tube placement. From this sample, 223 children (92.5%) were available for analysis because they were assessed at age 3 years and again at 9–11 years. This cohort closely represented the general population of children in the region of the country in which the study took place (50% Male, 82% Anglo-American, 15% African-American, 30% Medicaid).

This secondary data analysis was deemed exempt from human subjects review at Stanford University because the data were de-identified.

### Procedures

Table 1 summarizes the predictive measures collected at age 3 and outcome measures collected at age 9–11 years.

**Table 1:** **Summary of domains of function and measures to evaluate language, speech, cognition, and reading**.

**Age of testing**	**Domain**	**Measures**	**Description**
Age 3 years	Cognition	McCarthy Scales of Children's Ability (GCI)	Norm referenced test provides an age adjusted General Cognitive Index Score (GCI; Mean = 100, *sd* = 15)
	Receptive vocabulary	Peabody Picture Vocabulary Test-Revised (PPVT-R)	Norm referenced test in which children point to pictures that represent the spoken word. Number of correct responses calculated. Generates an age adjusted standard score (Mean = 100, *sd* = 15)
	Expressive vocabulary	Number of Different Words (NDW)	The number of different words that children utter during a recorded play session is counted
	Expressive syntax	Mean Length of Utterance-morphemes (MLUm)	The mean number of different morphemes per child's utterances during a recorded play session is calculated
	Speech	Percentage Consonants Correct-Revised (PCC-R)	Speech maturity is calculated by phonemic accuracy in relation to age expectations from a sample of speech recorded from a play session
Ages 9–11 years	Decoding	Woodcock Reading Mastery Tests Revised Norms Updated (WRMT R/NU) Basic Skills	Accuracy of single word and single pseudo word reading. Generates a standard score (Mean = 100, *sd* = 15)
	Reading comprehension	Woodcock Reading Mastery Tests Revised Norms Updated (WRMT R/NU) Passage Comprehension	Accuracy of completing the missing word in one to two sentences (Mean = 100, *sd* = 15)
	Oral reading fluency	Oral Reading Fluency Task	Three passages are supplied to children. The median number of words read aloud in one minute, per passage is calculated

#### Predictor variables assessed at age 3

Receptive Vocabulary was measured by the Peabody Picture Vocabulary Test-Revised (Dunn and Dunn, 1981). This standardized norm-referenced test requires no reading or writing, making it appropriate for preschool children. The PPVT-R has a mean of 100 and a standard deviation of 15. Reliability using coefficient alpha for children at age 3 years is 0.78.

Conversation analysis was used to assess expressive vocabulary, syntax, and speech sound maturity. For the conversation analysis, children were recorded for 15 min during informal play. Children were supplied a variety of toys including kitchen utensils, foods, and a playhouse. Caregivers and examiners were present with the child. Caregivers were instructed to play and speak to their child as if they were at home. The sessions were recorded for later analysis.

The number of different words (NDW) that the child used during the 15-min conversation was the measure of expressive vocabulary. There are no standardized norms for NDW as studies use different methods for collection and analysis.

Mean Length of Utterance in morphemes (MLUm) was used as a measure of expressive syntax. Complete intelligible utterances were recorded and analyzed using a computer-based program. The scores were calculated dividing the number of morphemes in all complete utterances by the number of utterances. Miller and Chapman predicted different norms and standard deviations for MLUm as a function of age in months. Based on their sample of 123 children, the mean ± SD MLUm of children 36 months of age is 3.16 ± 0.69 (Miller and Chapman, 1981).

Speech Sound Maturity was measured using The Percentage Consonants Correct-Revised (PCC-R), a measure developed by Shriberg and colleagues assesses phonemic accuracy in speech (Shriberg et al., 1997). The PCC-R penalizes consonant omissions and substitutions, but consonant distortions such as allophonic errors are scored as correct. This measure was particularly well-suited to the children at age 3 since distortions of speech are expected of typically developing children. The mean PCC-R value at 36 months was 86.6% (*SD* = 5.1%) (Campbell et al., 2007).

#### Mediator variable

Cognition served as the mediator variable and was measured using the McCarthy Scales of Children's Abilities (MSCA). This norm-referenced test generates a comprehensive score, the McCarthy Scales General Cognitive Index (MSGCI), calculated from the results of the Verbal, Perceptual, and Quantitative Performance Scores (McCarthy, 1972). The MSGCI has a mean score of 100 and a standard deviation of 15. The MSCA manual gives a split-half reliability of 0.93 for children.

#### Outcome variables assessed at age 9–11 years

The same children were retested between age 9 and 11 years for reading abilities. Variables included decoding, or basic single word reading skills, a composite score derived from reading words and pseudo-words; reading comprehension; and oral reading fluency.

Decoding was measured using The Woodcock Reading Mastery Test-Revised, Norms Updated (WRMT R/NU). The Basic Skills composite score was used to measure decoding skills. Word Identification tests children's ability to identify individual words. Word Attack asks children to apply phonemic rules and structural principles to pseudo words. The WRMT R/NU has a reliability test median (internal consistency) of.91 (Woodcock, 1987).

Reading comprehension was measured using The Woodcock Reading Mastery Test-Revised, Norms Updated (WRMT R/NU) Passage Comprehension score. Children read sentences silently and supply the word or words that complete meaning in the sentence.

Three passages were administered to children to test oral reading fluency. Children were given passages and instructed to read for 1 min per passage. The median of the words read in the three passages serves as the measure of oral reading fluency.

### Statistical analysis

We calculated descriptive statistics for all measures. We confirmed that all of the scores were normally distributed. Parametric correlations established whether predictor variables and mediator variable collected at age 3 years were associated with later reading outcome measures.

We then completed a mediation analysis, based on Hayes and Preacher (2013). Mediation analysis tests whether the effects of a given independent variable or variables on an outcome variable can be accounted for by its effect on an intermediate variable, or mediator, which in turn affects the outcome. In specific, the mediation analysis assesses the following associations: (1) whether the independent variables, also known as predictor variables contribute to the variance of the outcome variable (Total Effect), (2) whether the predictor variables contribute to the variance of the mediator and if the mediator contributes to the variance of the outcome measure (Indirect Effect), and (3) whether the predictor variables continued to predict the outcome variable with the mediator in the model (Direct Effect) (Field, 2013). Current versions of mediation analysis have moved beyond the causal steps approach (multi-step regression model), popularized by Baron and Kenny (1986), to a more robust approach that has more power and can quantify rather than simply infer the indirect effect (Hayes, 2009). We used the SPSS macros MEDIATE for mediation because they permit the analysis of multiple predictor variables on the outcome variable. We used a bootstrapping method with 10,000 re-samples to compute confidence intervals for the indirect effect and specifically to determine whether the mediator completely or partially mediated the effect of the predictor variables on the outcomes.

We performed this mediation analysis for each reading outcome to determine if the same associations governed each individual reading outcome.

## Results

### Correlation analyses

Table 2 shows the mean scores and standard deviations for all of the mediator, predictor, and outcome variables. Within the table, the standardized scores, available for cognition and receptive vocabulary at age 3 years and decoding and reading comprehension at age 9–11 years, all fell within the normal range. The mean MLUm for the sample was approximately one standard deviation above norms previously reported (Miller and Chapman, 1981).

**Table 2:** **Mean scores and standard deviation on all measures**.

	**Mean**	**Standard deviation (sd)**	**Number of subjects**
**AGE 3 YEARS**			
GCI	106.9	12.4	216
PPVT-R	101.8	15.1	211
NDW	179.9	36.3	211
MLUm	3.87	0.68	211
PCC-R	95.6	2.4	212
**AGE 9–11 YEARS**			
Basic skills	103.7	12.7	223
Passage comprehension	101.0	10.1	223
Oral reading fluency	110.7	35.9	222

*GCI, McCarthy Scales of Children's Abilities, General Cognitive Index; PPVT-R, Peabody Picture Vocabulary Test-Revised; NDW, Number of different words; MLUm, Mean Length of Utterance in morphemes; PCC-R, Percentage Consonants Correct-Revised*.

*Basic Skills from the Woodcock Reading Mastery Tests Revised Norms Updated*.

*Passage Comprehension from the Woodcock Reading Mastery Tests Revised Norms Updated*.

Table 3 shows the Pearson product moment correlations among the predictor variables at age 3 years. The predictor variables were all normally distributed and the assumption of lack of multicollinearity was met, using the Durbin-Watson statistic (all values were between 1.84 and 2.04). The table also includes the Pearson product moment correlations of the predictor variables and the mediator variable. As hypothesized and required for mediation analysis, all of these correlations were statistically significant.

**Table 3:** **Pearson moment correlations among preschool measures**.

**Measure**	**Correlations**	**Expressive vocabulary**	**Expressive syntax**	**Receptive vocabulary**	**Speech-sound maturity**	**Cognition**
Expressive Vocabulary	Pearson Correlation	1	0.589	0.224	0.203	0.260
	Sig. (2 tailed)		0.000	0.001	0.003	0.000
	*N*	211	211	210	211	205
Expressive Syntax	Pearson Correlation		1	0.200	0.128	0.333
	Sig. (2 tailed)			0.004	0.063	0.000
	*N*		211	210	211	205
Receptive Vocabulary	Pearson Correlation			1	0.394	0.706
	Sig. (2 tailed)				0.000	0.000
	*N*			222	211	215
Speech-sound Maturity	Pearson Correlation				1	0.283
	Sig. (2 tailed)					0.000
	*N*				212	206

Table 4 shows the Pearson moment correlations between the predictor and mediator variables at age 3 and the outcome variables at age 9–11 years. All predictor variables were significantly correlated with the later reading scores (*r* = 0.17–0.53, *p* < 0.012). Cognition was also significantly correlated with reading scores (*r* = 0.45 to 0.57, *p* < 0.0001).

**Table 4:** **Pearson moment correlations of preschool measures with school-aged reading measures**.

**Measure**	**Correlations**	**Decoding**	**Reading comprehension**	**Oral reading fluency**
Expressive vocabulary	Pearson correlation	0.173	0.338	0.326
	Sig. (2 tailed)	0.012	0.000	0.000
	*N*	211	211	211
Expressive syntax	Pearson correlation	0.270	0.351	0.374
	Sig. (2 tailed)	0.000	0.000	0.000
	*N*	211	211	211
Receptive vocabulary	Pearson correlation	0.385	0.528	0.457
	Sig. (2 tailed)	0.000	0.000	0.000
	*N*	222	222	221
Speech-sound maturity	Pearson correlation	0.338	0.340	0.338
	Sig. (2 tailed)	0.000	0.000	0.000
	*N*	212	212	212
Cognition	Pearson correlation	0.475	0.566	0.447
	Sig. (2 tailed)	0.000	0.000	0.000
	*N*	216	216	215

### Mediation analyses

**Figure 1:**
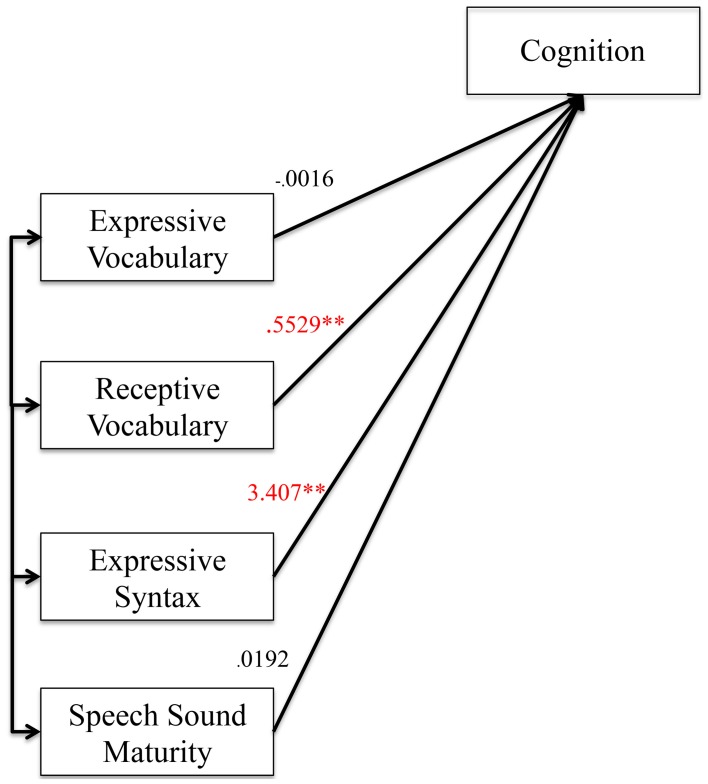
**Effect of the predictor variables on the mediator variable. Figure 1** shows the effects of preschool language and speech measures on cognition as the mediator variable. Unstandardized coefficients of correlations are included in model. Coefficients marked with a double asterisk are significant at the *p* < 0.01 level.

As a first step in the mediation analysis, we established that the predictor variables (preschool language and speech measures) were associated with the mediator variable (cognition). Figure 1 shows the coefficients and level of significance when the four predictors were simultaneously entered into the model predicting cognition. The overall model was highly significant with *R* = 0.73, *R*^2^ = 0.52, *F*_(4, 199)_ = 55.39, *p* < 0.0001.

Table 5 summarizes the mediation models of the three reading outcomes. In decoding, the variance accounted for by the predictors rose from *R* = 0.50 (*R*^2^ = 0.25) to *R* = 0.56 (*R*^2^ = 0.31) when cognition was included in the model as a mediator. In reading comprehension, the variance accounted for by the predictors rose from *R* = 0.61 (*R*^2^ = 0.38) to *R* = 0.65 (*R*^2^ = 0.43) when cognition was included in the model. In oral reading fluency, the variance accounted for by the predictors rose from *R* = 0.56 (*R*^2^ = 0.32) to *R* = 0.58 (*R*^2^ = 0.34) when cognition was included in the model.

**Table 5:** **Model summaries for total (without mediator) and direct (with mediator) effects of early skills on the components of reading**.

**Outcome**	***R***	***R^2^***	***F***	***df***	***p***
Decoding	0.50	0.25	16.76	4	0.0000
Decoding with cognition	0.56	0.31	17.99	5	0.0000
Reading comprehension	0.61	0.38	29.91	4	0.0000
Reading comprehension with cognition	0.65	0.43	29.57	5	0.0000
Oral reading fluency	0.56	0.32	22.94	4	0.0000
Oral reading fluency with cognition	0.58	0.34	20.33	5	0.0000

#### Decoding analyses

**Figure 2:**
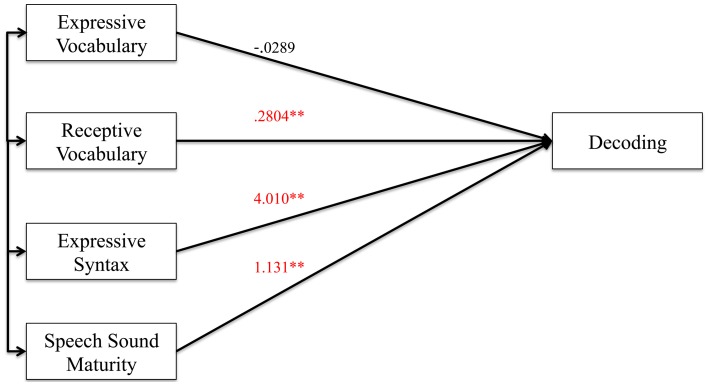
**Total effect model (without mediator) for decoding. Figure 2** shows the total effect model for preschool language and speech measures on decoding. Unstandardized coefficients of correlations are included in model. Coefficients marked with a double asterisk are significant at the *p* < 0.01 level.

**Figure 3:**
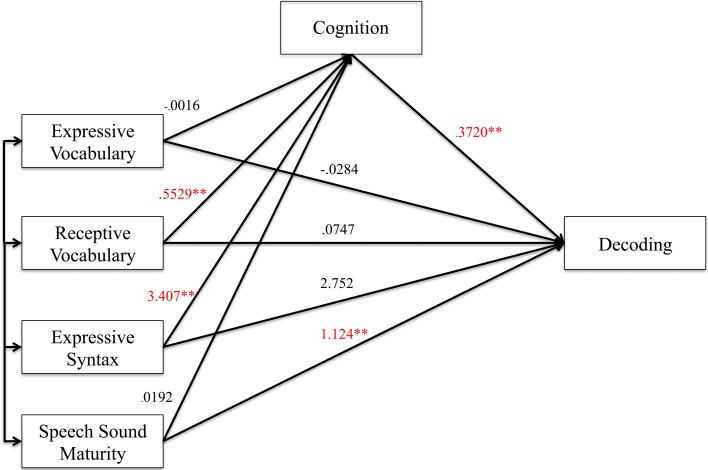
**Direct effect model (with mediator) for decoding. Figure 3** shows the direct effect model for preschool language and speech measures on decoding with cognition as the mediator. Unstandardized coefficients of correlations are included in model. Coefficients marked with a double asterisk are significant at the *p* < 0.01 level.

Figures 2, 3 show the results of the mediation analysis for decoding. In the total effect model without mediation (Figure 2), we found that receptive vocabulary (*p* < 0.0001), expressive syntax (*p* = 0.006) and speech sound maturity (*p* = 0.003) all had a significant positive association with decoding skills. In the direct effect model with cognition as a mediator (Figure 3), cognition was highly correlated with decoding (*p* < 0.0001). With cognition in the model, receptive vocabulary and expressive syntax were no longer significantly associated with decoding. Thus, cognition mediated the effects of receptive vocabulary and expressive syntax on decoding. Speech sound maturity made an independent contribution to decoding (*p* = 0.002), even when cognition was a mediator. These findings can be appreciated by inspection of the indirect effects from bootstrapping (Table 6). The confidence interval did not cross 0 for receptive vocabulary or expressive syntax, indicative of medication. However, the confidence interval did cross 0 for speech sound maturity indicating that the mediator did not significantly mediate the effects of this predictor on the outcome.

**Table 6:** **Indirect effect of predictor variables to decoding**.

	**Effect**	**SE (boot)**	**Lower limit confidence interval**	**Upper limit confidence interval**
Receptive vocabulary	0.2057	0.0590	0.1015	0.3330
Expressive syntax	1.268	0.5408	0.3889	2.479
Speech sound maturity	0.0072	0.1119	−0.2400	0.2117

*SE, Standard Error*.

#### Reading comprehension analyses

**Figure 4:**
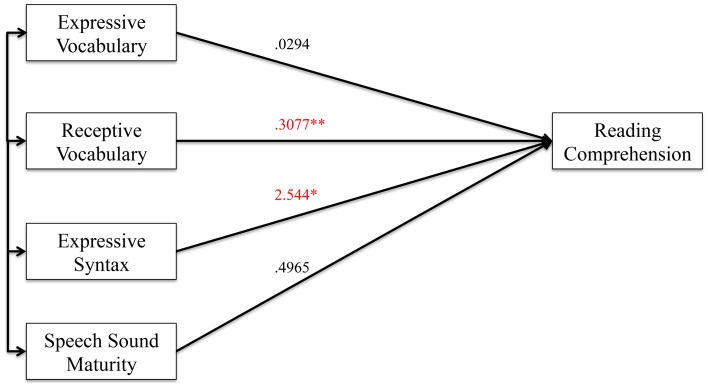
**Total effect model (without mediator) for reading comprehension. Figure 4** shows the total effect model for preschool language and speech measures on reading comprehension. Unstandardized coefficients of correlations are included in model. Coefficients marked with a single asterisk are significant at the *p* < 0.05 level. Coefficients marked with a double asterisk are significant at the *p* < 0.01 level.

**Figure 5:**
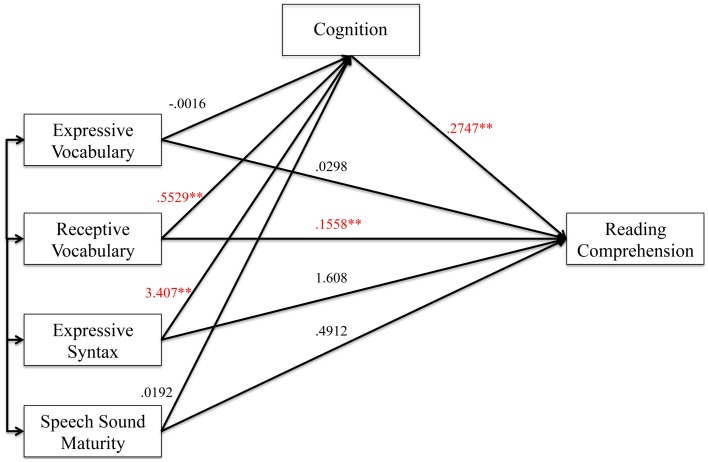
**Direct effect model (with mediator) for reading comprehension. Figure 5** shows the direct effect model for preschool language and speech measures on reading comprehension with cognition as the mediator. Unstandardized coefficients of correlations are included in model. Coefficients marked with a double asterisk are significant at the *p* < 0.01 level.

Figures 4, 5 show the results of the mediation analysis for reading comprehension. In the total effects model without mediation (Figure 4), we found that receptive vocabulary (*p* < 0.001) and expressive syntax (*p* = 0.016) positively correlated with reading comprehension. In the direct model with cognition as a mediator (Figure 5), expressive syntax was no longer significantly associated with comprehension. Thus, cognition mediated the effects of expressive syntax on comprehension. Receptive vocabulary remained significantly correlated with comprehension even when cognition served as a mediator in the model but the level of significance dropped slightly (*p* = 0.005). Thus, cognition partially mediated the association of receptive vocabulary on comprehension. This finding can also be appreciated from the indirect effects from bootstrapping (Table 7). The confidence interval does not cross 0 for receptive vocabulary, indicating partial mediation.

**Table 7:** **Indirect effect of predictor variables to reading comprehension**.

	**Effect**	**SE (boot)**	**Lower limit confidence interval**	**Upper limit confidence interval**
Receptive vocabulary	0.1519	0.0403	0.0801	0.2383
Expressive syntax	0.9359	0.3746	0.3126	1.782

*SE, Standard Error*.

#### Oral reading fluency analyses

**Figure 6:**
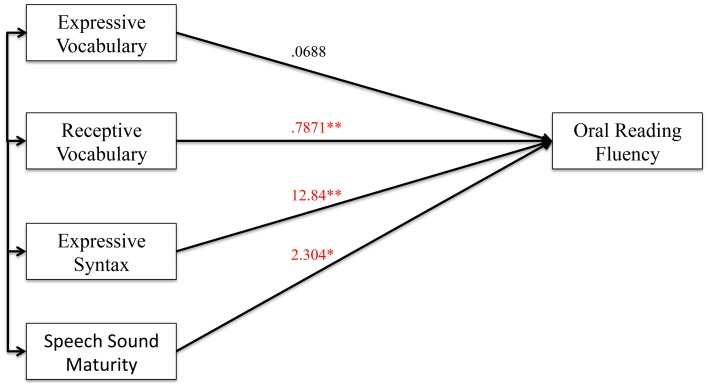
**Total effect model (without mediator) for oral reading fluency. Figure 6** shows the total effect model for preschool language and speech measures on oral reading fluency. Unstandardized coefficients of correlations are included in model. Coefficients marked with a single asterisk are significant at the *p* < 0.05 level. Coefficients marked with a double asterisk are significant at the *p* < 0.01 level.

**Figure 7:**
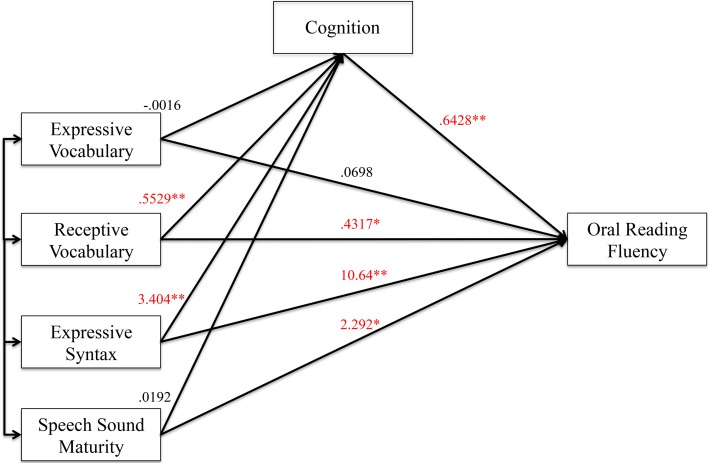
**Direct effect model (with mediator) for oral reading fluency. Figure 7** shows the direct effect model for preschool language and speech measures on oral reading fluency with cognition as the mediator. Unstandardized coefficients of correlations are included in model. Coefficients marked with a single asterisk are significant at the *p* < 0.05 level. Coefficients marked with a double asterisk are significant at the *p* < 0.01 level.

Figures 6, 7 show the results of the mediation analysis for oral reading fluency. In the total effects model without mediation (Figure 6), we found that receptive vocabulary (*p* < 0.0001), expressive syntax (*p* = 0.001), and speech sound maturity (*p* = 0.019) were significantly correlated to oral reading fluency. In the direct model with cognition as a mediator (Figure 7), receptive vocabulary (*p* = 0.036) and expressive syntax (*p* = 0.006) remained significantly correlated with oral reading fluency, but the level of significance dropped. Thus, cognition partially mediated the association of receptive vocabulary and expressive syntax on oral reading fluency. Speech sound maturity remained significantly correlated with oral reading fluency even when cognition served as a mediator in the model (*p* = 0.018). Thus, cognition did not mediate the effects of speech sound maturity on oral reading fluency. This finding can also be appreciated from the indirect effects from bootstrapping (Table 8). The confidence interval does not cross 0 for receptive vocabulary or expressive syntax, indicating partial mediation. The confidence interval does cross 0 for speech sound maturity, indicating no mediation effect.

**Table 8:** **Indirect effect of predictor variables to mediator and mediator to oral reading fluency**.

	**Effect**	**SE (boot)**	**Lower limit confidence interval**	**Upper limit confidence interval**
Receptive vocabulary	0.3554	0.1317	0.1051	0.6202
Expressive syntax	2.190	1.074	0.4416	4.660
Speech sound maturity	0.0124	0.2007	−0.4481	0.3717

*SE, Standard Error*.

## Discussion

In summary, in this longitudinal study, we found that all assessments of language skills, speech sound maturity, and cognition measured at age 3 years correlated significantly with decoding, reading comprehension, and oral reading fluency measured at age 9–11 years. Cognition at age 3 years was also associated with all reading measures at school age. Cognition mediated many of the associations. However, the effect of speech sound maturity made an independent contribution to decoding. The effect of receptive vocabulary on comprehension was only partially mediated by cognition, suggesting that it had an independent effect. The effects of receptive vocabulary and expressive syntax on oral reading fluency were also only partially mediated by cognition whereas the effect of speech sound maturity on oral reading fluency was not mediated by cognition. Thus, all three preschool skills contributed to the variance in oral reading fluency.

These results add to the previous literature about how early language and speech skills relate to later reading abilities, and may explain further why specific impairments to early language and speech could lead to later reading disability (Bishop and Adams, 1990; Rayner et al., 2001; Shaywitz et al., 2008). While all of the predictor and mediator variables correlated with all reading outcomes, cognition and receptive vocabulary had the highest correlations individually. We found that when cognition was placed in the mediation model, cognition mediated some and not all of the effects of the early language and speech on reading. Table 9 summarizes the findings.

**Table 9:** **Summary of associations of language and speech skills to reading outcomes with cognition as a mediator**.

**Outcome variable**	**Not a predictor, fully mediated by cognition**	**Predictor, partially mediated by cognition**	**Independent predictor, not mediated by cognition**
Decoding	Receptive vocabulary		Speech sound maturity
	Expressive syntax		
Reading Comprehension	Expressive syntax	Receptive Vocabulary	
Oral reading Fluency		Receptive vocabulary	Speech sound maturity
		Expressive syntax	

Similar to past studies, we found a high correlation of cognition with later reading. The correlations we found between cognition and reading were higher than those concurrent correlations found by Stanovich et al. within the first year of school (Stanovich et al., 1984). From this study, it can be hypothesized that language skills remain associated with reading despite the mediation from cognition because cognition and reading are highly related but not identical skills (Ferrer et al., 2007, 2010). In addition, language skills are also highly related to later reading ability. The magnitude of the mediation effect varied per domain of reading tested. Despite the mediation of cognition, several measures of language and speech remained independently associated with later reading outcomes.

Expressive vocabulary was weakly associated with later reading outcomes in simple correlations but not when other language and speech variables were included in a mediation analysis. This finding could be explained in several ways. Since the score for this measure was generated from a conversation analysis recorded during a play session in a new environment, the children's performance may not have been an accurate reflection of their true ability. Also, the test measure, Number of Different Words, was also correlated with each of the other measures. The effects of the other measures could therefore mask the effect of this variable on the reading outcomes.

Interestingly, speech sound maturity contributed to the variance in decoding and was not mediated by cognition. In decoding, phoneme to grapheme linkage is required. We suspect that speech sound maturity may predispose to early phonemic or phonological awareness that in turn correlates with reading (Storch and Whitehurst, 2002; NICHD Early Child Care Research Network, 2005). We unfortunately did not have a measure of phonological awareness at age 3 in this data set. Future studies should address how speech sound maturity measured at early ages influences speech perception and phonological awareness ability. Rvachew found that speech perception in pre-kindergarten explained variance in phonological awareness in kindergarten (Rvachew, 2006). It would be interesting to learn if phonological awareness mediates the effects of speech sound maturity on later reading outcomes. The National Early Literacy Panel emphasized the input of phonological awareness. However, phonological awareness is a nascent ability at age 3 years. Our data suggest that at this very young age, phonological awareness may not be a suitable target for intervention. However, speech sound maturity could be targeted in an early intervention program along with cognition to improve reading outcomes.

We also found that receptive vocabulary contributed to the variance in reading comprehension and was only partially mediated by cognition. These results are consistent with other studies that have found that vocabulary size is associated with reading comprehension (Muter et al., 2004; Cain and Oakhill, 2011). This finding was interesting because although vocabulary was highly correlated with cognitive scores at age 3 years, vocabulary nonetheless made an independent contribution to the variance in reading comprehension. This finding complements other studies that find that vocabulary size in the pre-school era accounts for unique variance in cognitive skills at age 8 (Marchman and Fernald, 2008). Other studies have found that cognitive ability and vocabulary are both predictors of later reading (Hammill and McNutt, 1981; Stanovich et al., 1984; Scarborough, 2001). This study adds that cognition partially mediates the effect of vocabulary on reading.

Speech sound maturity, syntax, and receptive vocabulary all contributed to oral reading fluency. Thus, oral reading fluency behaved like a composite of reading comprehension and decoding. Cognition partially mediated the effects of receptive vocabulary and syntax on oral reading fluency. As in decoding, speech sound maturity continued to contribute to the variance and was not mediated by cognition. The results are in agreement with researchers who have described oral reading fluency as the connection between decoding and reading comprehension (Pikulski and Chard, 2005).

This analysis had limitations. Analysis was limited to the measures administered to children, as this was a secondary analysis from a much larger study. The study sample was also limited to a single metropolitan area, which in turn affected the sample population demographics. Fortunately, the sample included African-American and European Americans and families of all socioeconomic strata.

This study has implications for developing future early interventions designed to improve reading outcomes. Early language and speech skills form the foundation for successful reading the later years (Rayner et al., 2001; Shaywitz et al., 2008). This current study implies that weak cognitive and language skills at age 3 years could be used to identify children at risk for delayed reading ability, reducing delay in identification of at-risk children until the elementary school years. The study also suggests that three domains should be the focus of such interventions to improve reading outcomes: cognition, speech sound maturity, and receptive vocabulary. Cognition might serve as a primary target because it is highly associated to all later reading outcomes, and partially mediates the effects of language on reading. Partial support for the importance of improving cognitive abilities comes from intervention studies that focused on inductive learning skills to improve both single word reading and word decoding scores (Papadopoulos et al., 2004; Das et al., 2008). The intervention that focused on phonics skills resulted only in improvements in reading decoding, whereas the broader intervention had additional effects. We hypothesize that targeting cognition in preschoolers may also improve reading comprehension and oral reading fluency at older ages. Based on the results of the current study, speech sound maturity would also be an appropriate target for intervention in preschoolers to improve future decoding and oral reading fluency. Mire and Montgomery have advocated implementing standardized, efficient interventions targeting speech sound deficiency, even at school age (Mire and Montgomery, 2009).

Future research should investigate the development of skills in the years between age 3 and 9–11 to elucidate how the associations and mediation effects change over time. Though several longitudinal studies have looked at early language skills in relation to later reading development (Dickinson and Tabors, 2001; Catts et al., 2005), it would be interesting to see how the skills and connections vary over time and if receptive vocabulary, speech sound maturity, and cognition continue to play an important role. In addition, future studies could look at how these early language and speech skills correlate to other reading domains such as rapid naming, phonological memory, language structure, and literacy knowledge (Scarborough, 2001; Lonigan and Shanahan, 2009).

In summary, we found that variation in reading ability at school ages has roots in early language and speech skills, though the associations varied as a function of the specific reading ability assessed at school age (Table 6). Cognition partially mediated the effects of language skills on reading comprehension and oral reading fluency. Cognition did not mediate the effects of speech sound maturity on decoding or oral reading fluency. The study provided clues to potential targets for early interventions designed to improve reading outcomes.

### Conflict of interest statement

The authors declare that the research was conducted in the absence of any commercial or financial relationships that could be construed as a potential conflict of interest.
